# Comprehensive Analytical Profiling for Sustainable Jewelry: A Multi-Technique Characterization of Essential Oil-Modified Rosin

**DOI:** 10.3390/mps9010020

**Published:** 2026-02-02

**Authors:** Dantong Huang, Kaewbucha Manus, Apiwathnasorn Chalisa, Tianyi Liu, Chuyun Yan, Yumeng Gu

**Affiliations:** 1 School of Arts and Design, Guangdong Eco-Engineering Polytechnic, Guangzhou 510520, China; dantong.hua@rmutto.ac.th; 2 Chakrabongse Bhuvanarth International for Interdisciplinary Studies (CBIS), Rajamangala University of Technology Tawan-ok, Bangkok 10400, Thailand; 3 Faculty of Fine and Applied Arts, Rajamangala University of Technology Thanyaburi, Khlong Luang, Pathum Thani 12110, Thailand; 4 College of Forestry and Landscape Architecture, South China Agricultural University, Guangzhou 510642, China

**Keywords:** rosin resin, essential oils, volatile organic compounds (VOCs), GC-Q/TOF-MS, LC-Q/TOF-MS, sustainable jewelry

## Abstract

Rosin, a renewable natural resin derived from pine trees, is a promising biomass material for sustainable product development, though its distinct intrinsic odor limits broader use. This study implemented a comprehensive analytical strategy to mitigate the odor by incorporating essential oils (EOs)—eucalyptus (EUC) and peppermint (MINT)—and to conduct a multi-analytical characterization of the modified rosin jewelry. By integrating complementary analytical techniques, including LC-Q/TOF-MS for non-volatile components and GC-Q/TOF-MS for volatile organic compounds (VOCs), we achieved a systematic chemical profiling of the materials. The core composition of rosin, dominated by abietic acid (>48%), remained stable across all samples. The incorporation of EOs significantly altered the VOC profiles: The total VOC signal (summed peak area) in MINT-modified rosin was 2.57-fold that of the EUC-modified sample, with monoterpenoids comprising 87.62% of its VOC signature. Eucalyptol and limonene were tentatively identified as the major components in the EUC sample, whereas menthone, menthol, and limonene predominated in the MINT sample. Multivariate statistical analysis highlighted that variations in specific VOCs—particularly menthone, menthol, eucalyptol, and allo-ocimene—were closely associated with differences in the scent profiles of each modification. This work illustrates how a multi-technique analytical strategy can both guide and assess the functional modification of sustainable biomass materials. The findings offer a practical approach to improving rosin’s functional properties while providing a methodological framework for the integrated characterization of complex biomaterials, supporting the development of eco-friendly products aligned with green chemistry and sustainable design principles.

## 1. Introduction

Rosin resin, a natural and biodegradable substance derived from pine tree exudates, exhibits excellent adhesion, flexibility, and rapid-drying properties [1]. As an environmentally friendly and renewable resource, it demonstrates considerable potential for applications in creative industries and jewelry making, owing to its favorable transparency, malleability, and distinctive luster with a warm texture [2]. Rosin is the brittle, translucent, non-volatile solid resin remaining after distilling volatile terpenes from oleoresin. It is often chemically modified to enhance its mechanical properties and compatibility, making it suitable for use in coatings, adhesives, and other industrial applications—such as improving the softness of varnishes, the film-forming ability of alkyd resins, and the viscosity and durability of polymerized rosin-based paints [3]. Currently, rosin and its derivatives serve as alternatives to petroleum-based synthetic resins and have been widely adopted in adhesives, coatings, and printing inks, thereby providing important pathways for developing novel eco-friendly polymeric materials [4,5,6,7].

However, the strong pine-like odor of rosin resin may be considered a notable drawback, necessitating technical approaches for its modulation. The incorporation of essential oils represents an effective physical modification strategy, which not only aims to introduce customizable aromatic properties into the product [8]—for instance, by imparting a pleasant fragrance to jewelry to enhance user experience—but may also modulate the release behavior of volatile organic compounds (VOCs) from rosin through molecular interactions. Nevertheless, systematic studies on rosin–essential oil composite systems, particularly regarding their impact on volatile and non-volatile components, remain scarce.

Essential oils (EOs), which are complex mixtures of natural volatile compounds extracted from aromatic plants, are widely utilized for their distinctive aroma, antimicrobial, and antioxidant activities. They are generally recognized as safe, non-toxic, and well-accepted by consumers [9]. EOs can also be incorporated as natural antimicrobial and antioxidant agents into films and coatings [10]. Despite their versatility and potential for improving the scent of rosin-based jewelry, the combined use of EOs with rosin resin has not been thoroughly investigated.

Headspace solid-phase microextraction coupled with gas chromatography–mass spectrometry (HS–SPME/GC–MS) is a well-established gold-standard method for analyzing VOCs, offering advantages such as simple sample preparation, high sensitivity, and reliable qualitative and quantitative capabilities [11]. This technique has been extensively applied to analyze volatile compounds in complex matrices, including food flavors [12], fragrances [13], plant aromas [14], and polymeric materials [15]. However, comparative and systematic studies on the volatile profiles of rosin resin blended with different essential oils are still lacking.

Therefore, this study aims to investigate the effect of various essential oils—such as peppermint and eucalyptus—on the volatile composition of rosin-based jewelry. By incorporating selected EOs into a rosin matrix to prepare jewelry samples, we employed liquid chromatography-quadrupole/time-of-flight mass spectrometry (LC-Q/TOF-MS) and gas chromatography-quadrupole/time-of-flight mass spectrometry (GC-Q/TOF-MS) to compare differences in non-volatile and volatile compound profiles between sample groups. The findings are expected to provide a scientific basis and formulation guidelines for developing odor-controllable, value-added, eco-friendly resin jewelry, thereby promoting the innovative application of natural materials in the fashion industry.

## 2. Materials and Methods

### 2.1. Materials and Sample Preparation

**Figure 1:**
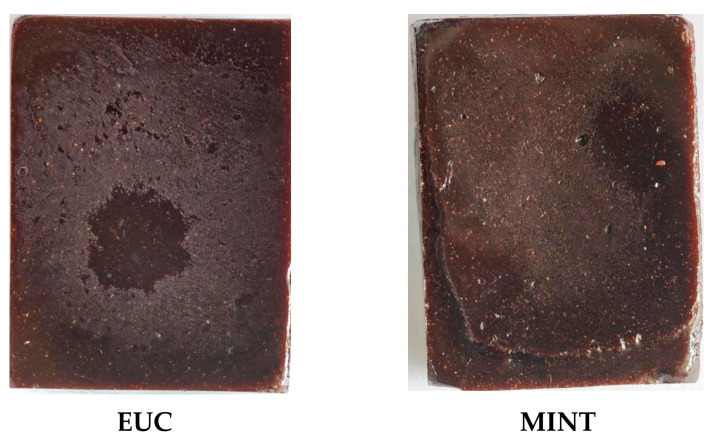
Rosin resin materials incorporated with natural essential oils. EUC denotes the rosin sample incorporating eucalyptus essential oil; MINT denotes the rosin sample incorporating peppermint essential oil.

Rosin resin (50.0 g), nanmu powder (5 g), and essential oil or inert substitute (8.0 g) were weighed into separate containers prior to use. To prepare the three sample types—EUC (eucalyptus essential oil), MINT (peppermint essential oil), and BLANK (matrix control)—a temperature-adjustable laboratory electric furnace was used to melt 50.0 g of rosin resin at 80 °C. Silica hardener and epoxy putty were then added, and the mixture was heated to 95 °C before emulsification with a JJ-1 electric stirrer until a homogeneous mixture was obtained. After confirming satisfactory emulsification, the blend was poured into molds and cooled under contamination-free conditions, followed by demolding after complete solidification. Final samples were shaped at 25 °C and exhibited softening after 8 h at 27 °C. For EUC and MINT, the 8.0 g oil phase comprised 100% pure eucalyptus or peppermint essential oil, respectively; for BLANK, an equal mass (8.0 g) of a high-boiling paraffin oil (boiling point >300 °C) was used to match the physical properties of the essential oils while contributing negligible volatile organic compounds under the analysis conditions. This design minimized differences in viscosity, hydrophobicity, and thermal behavior between samples, allowing VOC signals to be attributed more confidently to the essential oils rather than to matrix or additive emissions. Each formulation was prepared in triplicate (n = 3). Headspace GC–Q/TOF-MS analysis of the BLANK revealed a minimal background VOC profile, which was used to subtract matrix-derived contributions from the EUC and MINT datasets. Rosin resin and nanmu powder were obtained from Guangzhou Science and Trade Chemical Co., Ltd. (Guangzhou, China); both essential oils (100% purity) and paraffin oil were purchased from Yiqingtang Pharmaceutical Technology (Guangzhou) Co., Ltd. (Guangzhou, China). Samples containing eucalyptus and peppermint essential oils were labeled EUC and MINT, respectively, and the inert-oil control was labeled BLANK (Figure 1).

### 2.2. LC-Q/TOF-MS Analysis

Approximately 100 mg of powdered rosin sample was accurately weighed into a 15 mL centrifuge tube, and 5 mL of methanol was added. The mixture was ultrasonically extracted for 30 min, followed by centrifugation at 4000 r/min for 5 min. The supernatant was transferred to a 10 mL volumetric flask. The residue was re-extracted with 5 mL of methanol, and the supernatant was combined and diluted to volume with methanol. After mixing, the solution was filtered prior to analysis. A TripleTOF^®^ 5600+ high-resolution mass spectrometer (Sciex, Framingham, MA, USA) coupled with an LC-20XRD high-performance liquid chromatography system (Shimadzu, Japan) was used. Data acquisition was performed using Analyst TF 1.6 software (Sciex), and data processing was conducted using PeakView 2.0. Chromatographic separation was achieved on a Waters XBridge BEH C18 column (100 mm × 4.6 mm, 2.5 µm) with an injection volume of 10 µL, a flow rate of 0.4 mL/min, and a column temperature of 40 °C. The mobile phase consisted of acetonitrile and 0.1% formic acid in water. The gradient elution program was as follows: initial condition 95% aqueous phase (0.1% formic acid) and 5% acetonitrile; the acetonitrile proportion was linearly increased to 90% over 8 min, reached 100% at 13 min, and maintained until 15 min. The initial ratio was restored at 17 min and held until 20 min for column re-equilibration. Data were acquired in negative ion mode using an electrospray ionization (ESI) source of the TripleTOF^®^ 5600+ system (Sciex, Framingham, MA, USA). Full-scan TOF-MS data were collected in the m/z range of 50–2000 Da, and information-dependent acquisition (IDA) of MS/MS spectra was performed under the same mass range. The system was operated in high-sensitivity mode with dynamic background subtraction. Automatic mass calibration was performed after every five injections using a calibrant solution delivered at 0.5 mL/min. Each IDA cycle collected six scans. Ion source parameters were as follows: curtain gas (N_2_) 35 psi, nebulizer gas (N_2_) 50 psi, heater gas (N_2_) 50 psi, ion source temperature 450 °C, orifice voltage 4500 V, declustering voltage −90 V, and collision energies of −15, −35, and −55 eV. Detection was performed in TOF-IDA-MS mode. All samples were analyzed in triplicate.

### 2.3. GC-Q/TOF-MS Analysis

Analysis was performed using an Agilent 7200B gas chromatograph–quadrupole/time-of-flight mass spectrometer (GC-Q/TOF-MS) (Agilent Technologies, Santa Clara, CA, USA) equipped with a CTC autosampler (CTC Analytics AG, Zwingen, Switzerland) and a split/splitless inlet (Agilent Technologies, Santa Clara, CA, USA) fitted with a deactivated quartz single-taper liner (2 mm i.d., 78 mm length) (Agilent Technologies, Santa Clara, CA, USA) capable of withstanding temperatures up to 300 °C. Approximately 0.10 g of rosin sample (accurate to 0.01 g) was placed in a 20 mL headspace vial, (Agilent Technologies, Santa Clara, CA, USA) equilibrated at 80 °C for 30 min, and volatile compounds were adsorbed onto a 65 µm DVB/PDMS fiber (Supelco, Bellefonte, PA, USA) at 80 °C for 30 min. Thermal desorption of the fiber was performed in the GC split inlet, which was maintained at 260 °C throughout the process to ensure complete vaporization of analytes. Desorption was carried out in split mode with a split ratio of 10:1, and the fiber remained in the inlet for 5 min to allow complete transfer of volatiles onto the column. Following desorption, the inlet temperature was set to 250 °C for the GC run to maintain consistency with the programmed temperature gradient.

GC-Q/TOF-MS operating conditions were as follows: carrier gas, high-purity helium at a constant flow rate of 1.2 mL/min; transfer line temperature, 280 °C; capillary column, DB-5MS (30 m × 0.25 mm, 0.25 µm film thickness); full-scan acquisition mode with a mass range of *m*/*z* 40–550. The GC temperature program was: initial temperature 40 °C held for 1 min, increased to 180 °C at 2 °C/min and held for 1 min, then raised to 220 °C at 1 °C/min and held for 1 min, and finally increased to 280 °C at 5 °C/min and held for 2 min. To obtain retention indices for compound identification, a series of n-alkanes (C9–C15) was analyzed under identical GC-MS conditions. Retention indices were calculated based on the Kovats method. Putative compound identification was based on comparison of mass spectra with the NIST library (match threshold >80) combined with agreement of experimental retention indices with literature values. The VOC analysis selectively focused on VOCs originating from the two added essential oils. To achieve this, profiles of the EUC and MINT samples were compared against the unmodified rosin (BLANK) to distinguish and subsequently analyze only those compounds derived from eucalyptus or peppermint oils. A compound was retained for further analysis only if it was detected in the EUC and/or MINT sample, and either was absent in BLANK or exhibited a signal intensity at least 10% higher (*p* < 0.05) than that in BLANK. All analyses were performed in triplicate. Relative abundances of compounds were calculated based on peak areas. Given that HS-SPME peak areas are strongly influenced by matrix effects and fiber partitioning behavior, all quantitative comparisons presented in this work are semi-quantitative and expressed solely as relative peak areas.

### 2.4. Statistical Analysis

Student’s *t*-tests were conducted using built-in functions in R version 4.4.1. Volcano plots were generated using the “MetaboAnalystR” package (V4.0) to visualize differentially abundant volatile organic compounds (VOCs), with significance thresholds set at *p* < 0.05 and |Log_2_FC| > 1. Hierarchical cluster analysis (HCA) and heatmaps were produced using the “pheatmap” package. Orthogonal projections to latent structures-discriminant analysis (OPLS-DA) and variable importance in projection (VIP) scores were calculated using the “MetaboAnalystR” package (V4.0). The OPLS-DA model was validated by permutation testing with 1000 iterations to assess robustness. VOCs with VIP > 1 were considered important differential compounds. The intersection of VOCs meeting both the t-test threshold (*FDR* < 0.05 and |Log_2_FC| > 1) and VIP > 1 was identified as the most critical set of differential compounds between sample groups.

## 3. Results

### 3.1. Comparative Analysis of Non-Volatile Compounds by LC-Q/TOF-MS

**Figure 2:**
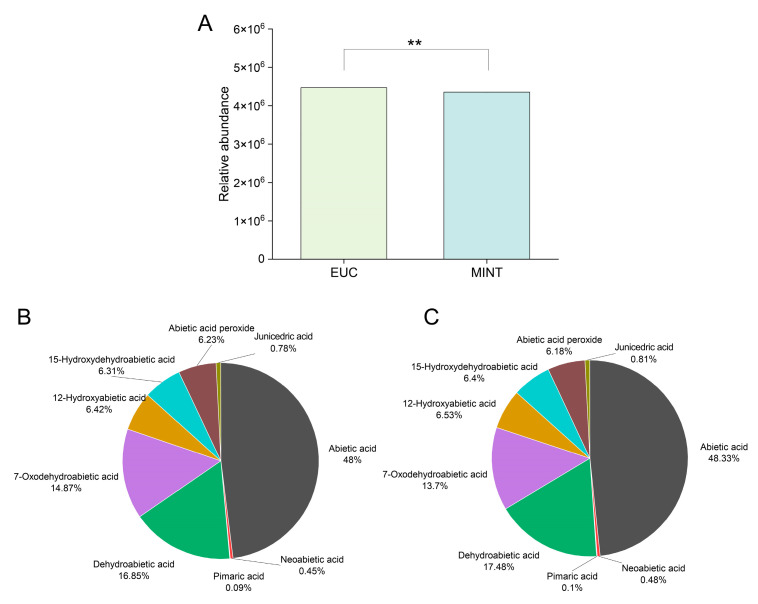
Analysis of non-volatile compound composition in EUC and MINT samples using LC–Q/TOF–MS. (**A**) Comparative analysis of total relative compound abundance. ** indicates a statistically significant difference at *p* < 0.01 by Student’s *t*-test. (**B**,**C**) Relative abundance distribution of nine major compounds in EUC and MINT, respectively.

The non-volatile compounds in the two rosin resin samples were first analyzed using LC-Q/TOF-MS. A total of nine characteristic rosin resin compounds were identified, including abietic acid, neoabietic acid, pimaric acid, dehydroabietic acid, 7-oxodehydroabietic acid, 12-hydroxyabietic acid, 15-hydroxydehydroabietic acid, abietic acid peroxide, and junicedric acid. Among these, abietic acid exhibited the highest relative abundance, whereas pimaric acid showed the lowest. Overall, the total relative abundance of the nine compounds was significantly higher in EUC than in MINT, though the relative abundance was only 0.87% greater in EUC (Figure 2A). At the individual compound level, only 7-oxodehydroabietic acid and abietic acid peroxide were more abundant in EUC than in MINT; the other seven compounds were slightly lower in EUC. In terms of compositional proportion, abietic acid constituted the majority in both samples, exceeding 48%, followed by dehydroabietic acid (16.85% in EUC, 17.48% in MINT) and 7-oxodehydroabietic acid (14.87% in EUC, 13.70% in MINT) (Figure 2B,C). In contrast, junicedric acid, neoabietic acid, and pimaric acid each accounted for less than 1% in both samples. Statistical analysis indicated no significant differences in the relative contents of the nine non-volatile compounds between the two samples (Table S1). In summary, the addition of eucalyptus or peppermint essential oil did not significantly alter the composition of non-volatile compounds in the rosin resin, and the relative abundance and distribution of compounds were highly similar between the two samples.

### 3.2. Comparative Analysis of Volatile Compounds by GC-Q/TOF-MS

**Figure 3:**
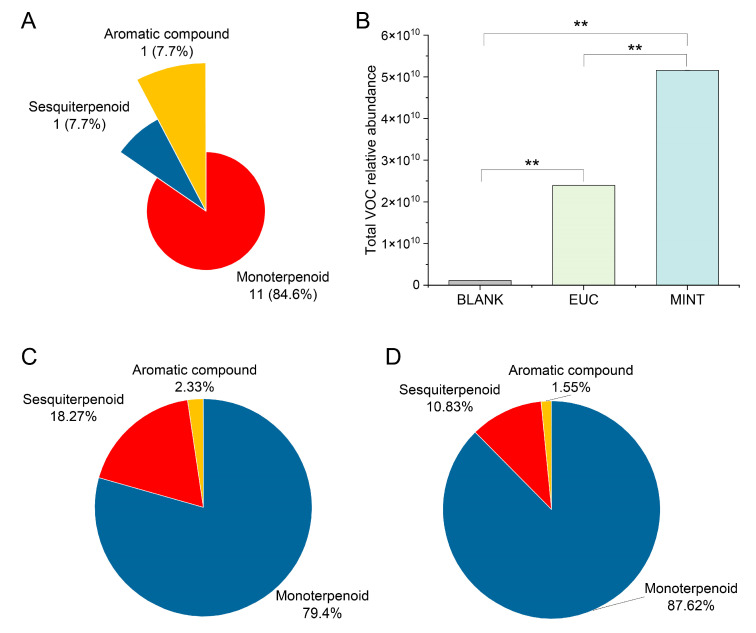
Composition of essential-oil-derived volatile organic compound (VOC) composition in different samples as determined by GC–Q/TOF–MS. (**A**) Distribution of identified VOCs among chemical categories. (**B**) Comparison of total relative VOC abundance among BLANK, EUC and MINT samples. ** denotes significant difference at *p* < 0.01 (Student’s *t*-test). (**C**,**D**) Relative abundance profiles of major VOC categories in EUC and MINT, respectively. EUC denotes the rosin sample incorporating eucalyptus essential oil; MINT denotes the rosin sample incorporating peppermint essential oil.

Volatile organic compounds (VOCs) in the two samples were further analyzed using GC-Q/TOF-MS. A rosin resin sample without added essential oil was used as the control to exclude VOCs unrelated to the two plant essential oils. Representative total ion current (TIC) chromatograms of the BLANK, EUC, and MINT samples are shown in Figure S1. Numerous peaks observed in EUC and MINT were absent in the BLANK. A total of 13 essential-oil-derived VOCs were identified, comprising 11 monoterpenoids, one sesquiterpenoid, and one aromatic compound (Figure 3A; Table S2). Among these, eleven VOCs (α-pinene, 3-carene, α-phellandrene, γ-terpinene, limonene, eucalyptol, fenchone, menthone, menthol, estragole, caryophyllene) were putatively identified by both mass spectrometry (MS) and retention index (RI) matching, while two VOCs (allo-ocimene and trans-ocimenol) were tentatively identified by MS alone (Table S2; Figure S2). Notably, the BLANK sample released only one of these compounds—caryophyllene, a sesquiterpenoid.

The total GC-MS signal intensity for all detected VOCs differed significantly between samples: the summed signal in EUC was 6.43-fold higher than in BLANK, and in MINT 16.54-fold higher than in BLANK; correspondingly, the total VOC signal in MINT was 2.57-fold that in EUC (Figure 3B). These results demonstrate that incorporating plant essential oils significantly enhances VOC emissions from rosin resin jewelry. We first compared the relative peak areas of caryophyllene, which was detected in all three samples (Figure S3). While the BLANK sample released a baseline level of caryophyllene—confirming its natural origin from the rosin itself—both EUC and MINT samples showed significantly higher levels (*p* < 0.01). This confirms a dual origin for this compound: a baseline from the rosin matrix, plus a substantial additive contribution from the essential oils. Despite its presence in the blank, caryophyllene was retained as a key analyte because the significant, oil-dependent increase in its emission provides a measurable signal to evaluate the essential oil contribution and to differentiate between the two scented formulations. Its status as a common constituent of both eucalyptus and peppermint oils further supports its relevance for comparative analysis.

Since BLANK released only one VOC (caryophyllene), subsequent analyses focused on comparing EUC and MINT to elucidate VOC differences resulting from the incorporation of different essential oils. The relative signal intensities of each VOC category were also higher in MINT than in EUC: monoterpenoid, sesquiterpenoid, and aromatic compound signals were 2.84-fold, 1.53-fold, and 1.71-fold those in EUC, respectively. The VOC composition also differed markedly between samples. Although monoterpenoids were dominant in both, the proportion of monoterpenoids in the MINT sample (87.62%) was substantially higher than that in the EUC sample (79.40%). Conversely, the proportions of sesquiterpenoid and aromatic compounds were higher in EUC than in MINT (Figure 3C,D). Additionally, in the EUC sample, eucalyptol and limonene were the major components, each accounting for more than 30% of the total VOC profile; in the MINT sample, menthone, menthol, and limonene were predominant, each exceeding 22% relative abundance. These compositional differences may contribute to distinct sensory properties between the two products.

### 3.3. Hierarchical Clustering Analysis Based on VOCs

**Figure 4:**
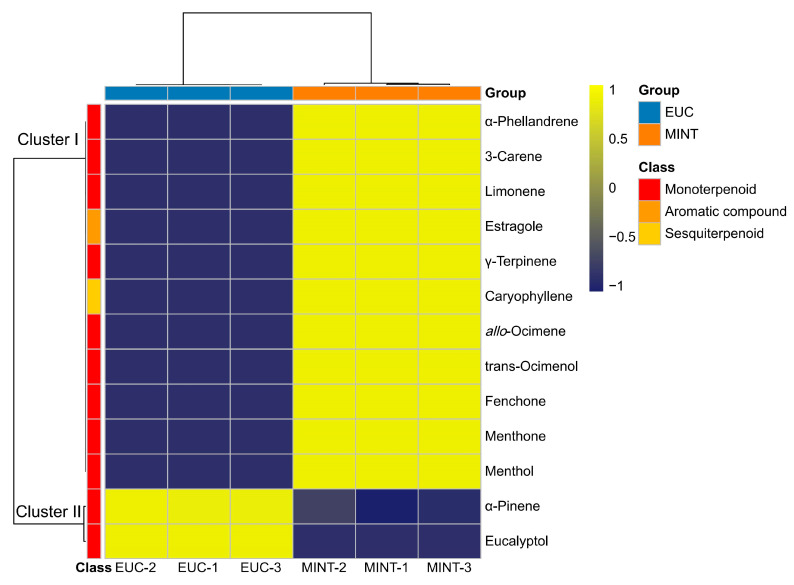
Hierarchical clustering heatmap of 13 VOCs in EUC and MINT samples. EUC denotes the rosin sample incorporating eucalyptus essential oil; MINT denotes the rosin sample incorporating peppermint essential oil.

To further investigate differences in specific VOCs, hierarchical clustering analysis (HCA) was performed. The three replicates of each sample clustered together, indicating good reproducibility. The VOC clustering analysis revealed that the 13 VOCs could be divided into two clusters (Figure 4). Cluster 1 contained 12 VOCs (nine monoterpenoids, one sesquiterpenoid, one aromatic compound), all of which showed higher relative abundance in MINT. Cluster 2 consisted of two VOCs (two monoterpenoids) that were more abundant in EUC. These results indicate that the higher total VOC signal (summed peak area) in MINT is attributable to a broad range of VOCs.

### 3.4. Identification of Differential VOCs via t-Test and VIP Values

**Figure 5:**
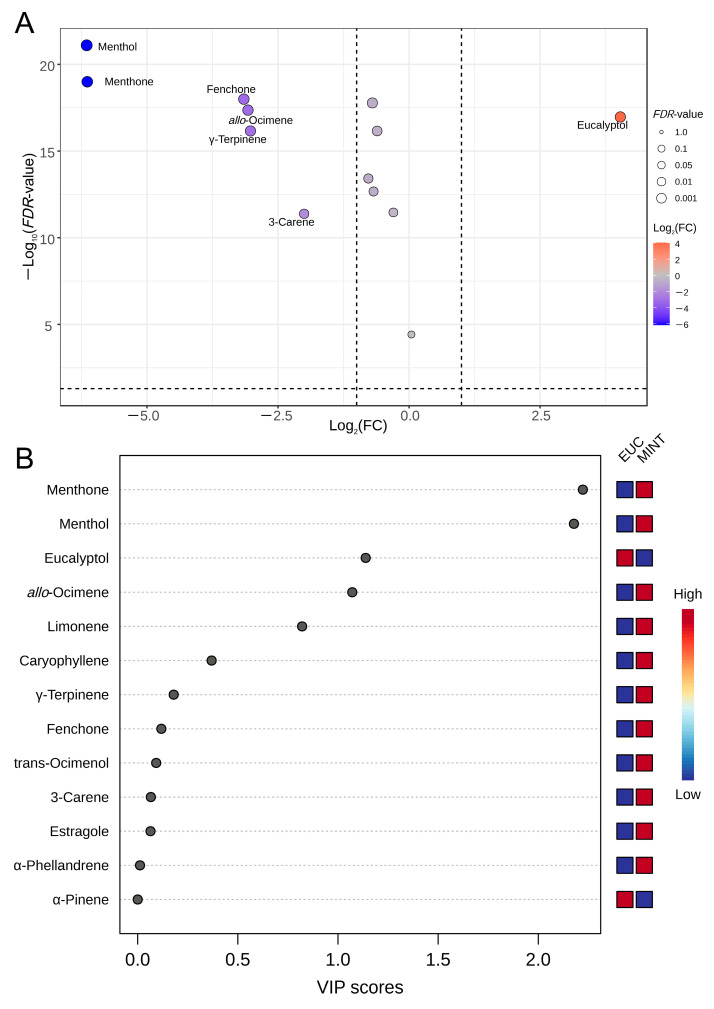
Identification of differential metabolites between EUC and MINT. (**A**) Volcano plot based on Student’s *t*-test. (**B**) Thirteen VOCs ranked by variable importance in projection (VIP) scores from OPLS–DA analysis.

To identify VOCs with significant differences between the EUC and MINT samples, a *t*-test was conducted on the 13 VOCs (Table S3). The analysis revealed seven VOCs that differed significantly (*FDR* < 0.05, |Log_2_FC| > 1). Among these, six VOCs—menthol, menthone, fenchone, *allo*-ocimene, γ-terpinene, and 3-carene—were more abundant in MINT (Figure 5A). Only one VOC (eucalyptol) was more abundant in EUC (Figure 5A). An orthogonal projections to latent structures-discriminant analysis (OPLS-DA) was subsequently conducted, yielding *R*^2^*Y* = 0.476 and *Q*^2^ = 0.41, indicative of reasonably good predictive capability. Variable importance in projection (VIP) scores identified four VOCs with VIP > 1, including menthone, menthol, eucalyptol, and *allo*-ocimene, highlighting their major contribution to group separation (Figure 5B; Table S4). However, permutation testing returned a *p*-value of 0.091 (>0.05), likely reflecting the limited sample size. Consequently, we integrated *FDR*, |log_2_FC|, and VIP criteria to determine the VOCs most strongly associated with the differences between the two sample groups.

**Figure 6:**
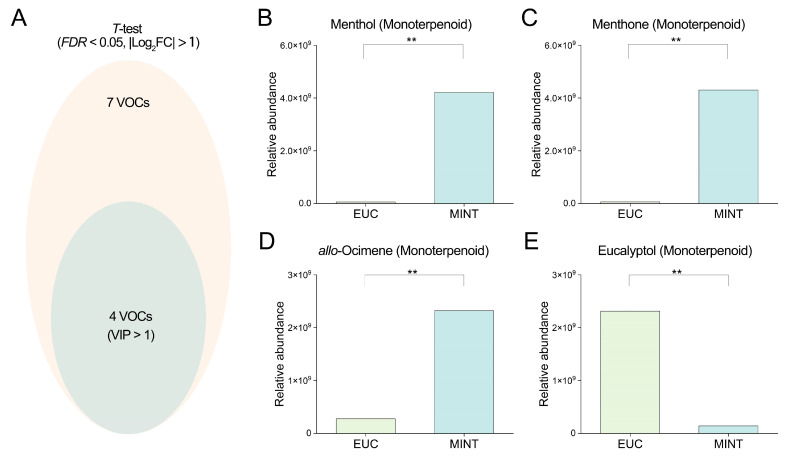
Screening of VOC candidates based on the intersection of *t*-test and VIP value criteria. (**A**) Venn diagram illustrating the overlap between statistically significant and biologically relevant VOCs. (**B**–**E**) Comparative analysis of the relative abundances of menthol, menthone, allo-ocimene, and eucalyptol in EUC and MINT. ** indicates *p* < 0.01 (Student’s *t*-test). EUC denotes the rosin sample incorporating eucalyptus essential oil; MINT denotes the rosin sample incorporating peppermint essential oil.

Integrating the results from the t-test and VIP scores, the intersection of the seven compounds found to be significant in the *t*-test and the four compounds with VIP > 1 yielded four overlapping compounds—menthone, menthol, eucalyptol, and allo-ocimene—which were thus considered the most differentiating VOCs between the samples (Figure 6). We further extracted these four selected compounds to compare their relative abundance ratios between the two sample groups. The summed GC-MS signal intensities of menthol, menthone, and allo-ocimene in the MINT sample were 70.05-fold, 70.49-fold, and 8.42-fold, respectively, of those in the EUC sample. Conversely, the signal intensity of eucalyptol in the EUC sample was 16.35-fold that in the MINT sample. These compounds, showing the most pronounced differences, are likely linked to the distinct aromatic properties of the two products.

## 4. Discussion

Introducing GC-MS into the study of volatiles in jewelry design contributes to the scientific evaluation of VOC emissions from accessories and supports the development of scented jewelry. Gas chromatography–mass spectrometry is widely recognized as a gold-standard method for VOC detection due to its powerful qualitative and quantitative capabilities [16], and it has been applied across numerous fields. Notably, several studies have successfully employed GC-MS to analyze volatiles from jewelry, such as identifying adhesive compounds in ancient artifacts [17] and detecting hazardous substances in imitation jewelry [18]. For instance, Cilová et al. used FTIR and GC-MS to identify birch bark tar as an adhesive in La Tène period jewelry, thereby expanding knowledge about historical jewelry production techniques [19]. In another study, a reliable GC-MS method was developed to determine 14 phthalic acid esters (PAEs) in imitation jewelry [18]. In the present work, GC-Q/TOF-MS was used to analyze VOCs from two types of rosin-based jewelry materials incorporating natural essential oils. A total of 13 VOCs were putatively identified, and multivariate analysis highlighted the main differences in VOC profiles between the two products. This approach provides a methodological reference for systematically evaluating the VOC characteristics of scented jewelry. In the future, VOC quality assessments can be efficiently extended to more formulations, laying a foundation for developing a wider range of fragrant accessories.

The incorporation of essential oils into rosin-based materials extends the functionality of traditional rosin jewelry beyond its inherent tackiness and renewability. In the context of scented jewelry, the appeal partly lies in the documented properties of essential oils, which are known to exert various physiological effects, such as mood regulation, stress reduction, alleviation of sleep disorders, and modulation of cognitive functions [20]. Peppermint oil, with its notable antibacterial and antioxidant properties, is considered a promising natural preservative, flavoring, insecticide, cooling agent, and herbal remedy [21,22]. Eucalyptus oil, another essential oil with broad bioactivities, exhibits antimicrobial, antiseptic, antioxidant, and therapeutic effects, and is used in wound healing, insect repellency, weed control, and the production of perfumes and soaps [23]. Therefore, the rationale for this study was to explore whether the characteristic volatile compounds of these oils could be effectively integrated into a rosin matrix, thereby imparting a sustained aromatic profile and, potentially, a functional dimension inspired by their reported bioactivities. Essential oils rich in volatile oxygenated monoterpenes can transform the odor profile of rosin, which is otherwise largely non-aromatic, thereby creating scented accessories with added sensory and potentially functional value. Our GC-Q/TOF-MS analysis putatively identified characteristic compounds associated with each essential oil in the corresponding rosin jewelry: eucalyptol was detected in the EUC sample [24]. In addition, peppermint oil is generally reported to be rich in L-menthone and levomenthol [25,26]. While our data confirm the presence of menthol, the non-chiral GC-MS method employed here cannot distinguish between enantiomers; therefore, chiral separation using a chiral stationary phase column is required in future work to confirm the specific stereoisomers. These fragrance-related compounds, along with their associated scent profiles, can be incorporated into jewelry products, thereby expanding product diversity. This result provides a reference for the further application of plant essential oils in the development of sustainable jewelry products.

The total VOC signal in the MINT sample was 2.57-fold that in the EUC sample, a difference primarily attributable to the distinct chemical compositions of the two essential oils. The MINT formulation contained a higher proportion of highly volatile oxygenated monoterpenoids (e.g., menthone and menthol), which are more readily detected under identical headspace sampling conditions compared with the predominantly monoterpenoid eucalyptol in EUC. Although peppermint oil displays a slightly higher polarity due to its greater fraction of oxygenated terpenes, both oils are largely non-polar; therefore, polarity differences are unlikely to be the main driver of the observed VOC abundance disparity. Instead, the higher content of light, volatile oxygenated compounds in MINT accounts for the stronger VOC signal. Variations in instrumental response to compounds of differing volatility may also contribute to the quantitative difference. These results indicate that the volatile characteristics of the essential oils are effectively retained in the rosin matrix, enhancing the olfactory attributes of the jewelry. Moreover, LC-Q/TOF-MS analysis confirmed that the addition of essential oils did not significantly alter the fundamental composition of the rosin resin. However, the longevity of these volatile components and their appropriate dosage for potential health benefits require further investigation.

It is also important to consider potential risks associated with dermal exposure. Some constituents of essential oils, such as eucalyptol [27,28], can cause skin irritation or allergic reactions in sensitive individuals, particularly at elevated concentrations or upon prolonged contact. Therefore, the incorporation of essential oils into rosin jewelry should be accompanied by safety assessments, including patch testing and evaluation of cumulative dermal effects, to ensure consumer well-being, especially for vulnerable groups such as children and individuals with pre-existing dermatological conditions.

From a broader sustainability perspective, the conventional jewelry supply chain—particularly the mining and processing of precious metals and gemstones—is a significant contributor to local environmental pollution and resource depletion [29]. In addition, the presence of hazardous elements (e.g., Pb, Cd, and Hg) in low-cost jewelry poses considerable health risks [30]. Consequently, the development of green and sustainable jewelry alternatives has become an important industry trend. The rosin-based jewelry material developed in this study is derived mainly from natural plant sources, and the incorporated essential oils contain numerous bioactive compounds, giving these products considerable potential as eco-friendly fashion accessories.

Although the present study systematically characterized the volatile and non-volatile compounds in the two jewelry formulations using GC-Q/TOF-MS and LC-Q/TOF-MS, such compositional data alone are insufficient to support health-related claims; therefore, further safety assessments are necessary. More importantly, as this represents a preliminary investigation into applying plant essential oils in sustainable jewelry design, the following research directions are proposed for future work: (1) designing additional formulations with varying essential oil concentrations; (2) expanding the range of plant essential oils investigated; and (3) conducting further physicochemical and stability testing of the composite materials. Critically, comprehensive safety evaluations must be prioritized, including skin contact tests, determination of safe inhalation levels for volatile compounds, and assessments of effects on vulnerable populations (e.g., pregnant women, children, and the elderly). These studies will help to ensure the development of truly healthy, environmentally sound, and distinctive jewelry products.

## 5. Conclusions

This study demonstrates that the incorporation of nature-derived essential oils—eucalyptus and peppermint—results in distinct volatile profiles and scent characteristics in rosin-based jewelry, while leaving its fundamental non-volatile composition largely unchanged, as shown by GC-Q/TOF-MS and LC-Q/TOF-MS analyses. The observed differences in VOC patterns between the two formulations are associated with notable differences in the semi-quantitative abundance of putatively identified characteristic compounds, such as eucalyptol in the EUC sample and menthol and menthone in the MINT sample, which were putatively identified and compared semi-quantitatively based on GC-Q/TOF-MS peak areas. These results illustrate how the choice of essential oil can be used to tailor the odor profile of a bio-based resin material, as revealed by comparative analytical measurements. The study also shows the utility of combining advanced analytical techniques with multivariate statistical analysis for characterizing complex mixtures in composite natural materials. The methodology established here provides a framework for the systematic analytical characterization of future scented bio-resin formulations.

## Data Availability

The original contributions of this study are provided in the Supplementary Materials. Further inquiries should be directed to the corresponding author.

## Supplementary Materials

The following supporting information can be downloaded at https://www.mdpi.com/article/10.3390/mps9010020/s1, Figure S1: Total ion current (TIC) chromatograms of BLANK, EUC and MINT samples obtained by GC-Q/TOF-MS. Peaks marked with arrows correspond to representative volatile organic compounds (VOCs) derived from the respective plant essential oils. BLANK denotes the rosin resin sample without added essential oil; EUC denotes the rosin sample incorporating eucalyptus essential oil; MINT denotes the rosin sample incorporating peppermint essential oil; Figure S2: Mass spectra and chemical structures of the 13 VOCs identified through mass spectral library matching and retention index comparison; Figure S3: Comparison of relative abundance of caryophyllene among BLANK, EUC, and MINT Samples. ** indicates *p* < 0.01 (Student’s *t*-test); Table S1: Summary of nine compounds identified by LC-Q/TOF-MS and their significant difference analysis between EUC and MINT; Table S2: List of 13 VOCs Detected by GC-Q/TOF-MS; Table S3: Comparison of relative signal intensities for 13 VOCs between EUC and MINT samples; Table S4: Summary of VIP values for the 13 VOCs derived from OPLS-DA.
