# Supplementary material for: Comprehensive Analytical Profiling for Sustainable Jewelry: A Multi-Technique Characterization of Essential Oil-Modified Rosin
Source: Methods Protoc. 2026 Feb 2;9(1):20. doi: 10.3390/mps9010020 (PMC12921789; doi:10.3390/mps9010020)
Supplement: Supplementary file 1 [file mps-09-00020-s001.zip › Figure S1-S3 R4.pdf]

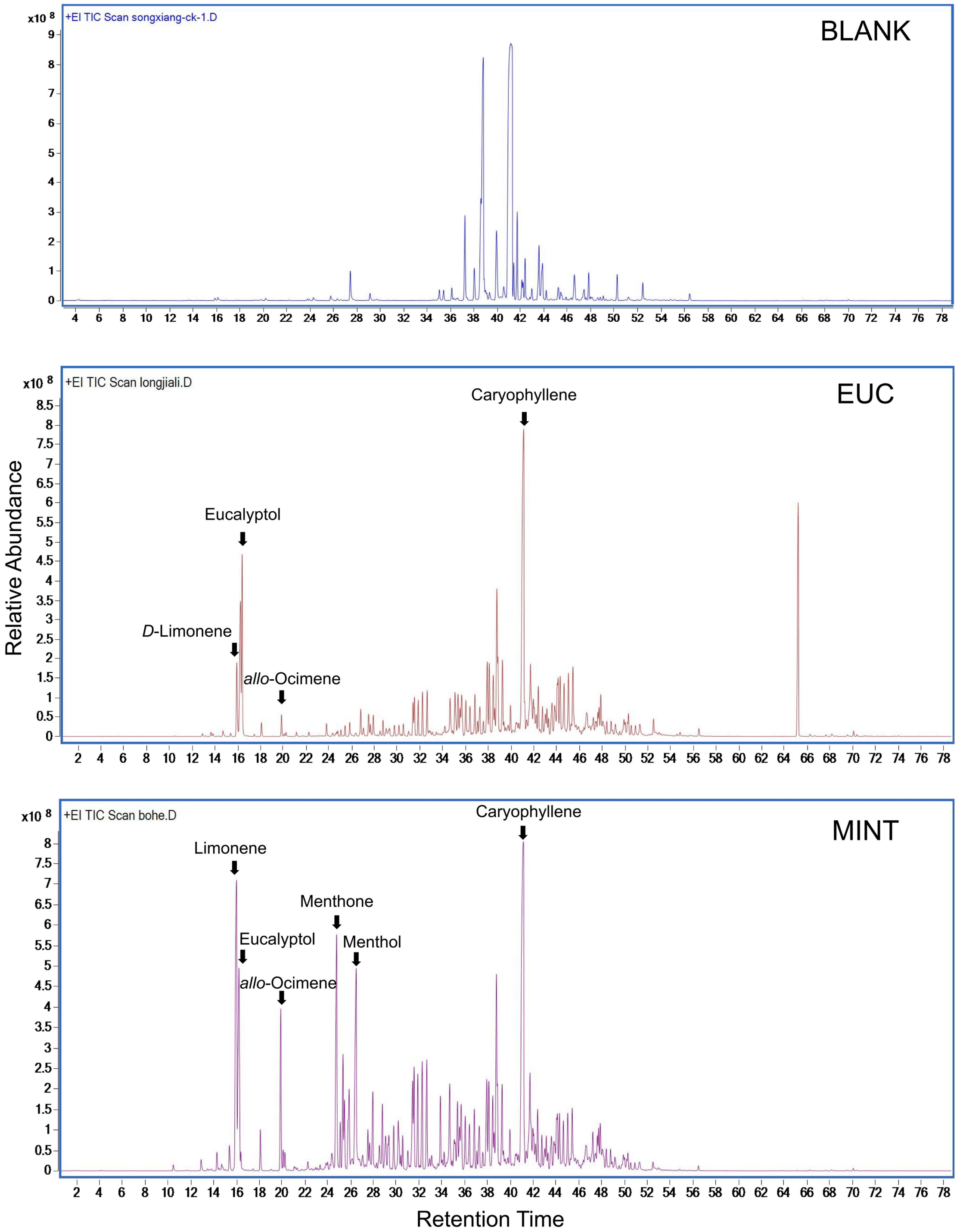

Figure S1. Total ion current (TIC) chromatograms of BLANK, EUC and MINT samples obtained by GC-Q/TOF-MS. Peaks marked with arrows correspond to representative volatile organic compounds (VOCs) derived from the respective plant essential oils. BLANK denotes the rosin resin sample without added essential oil; EUC denotes the rosin sample incorporating eucalyptus essential oil; MINT denotes the rosin sample incorporating peppermint essential oil.

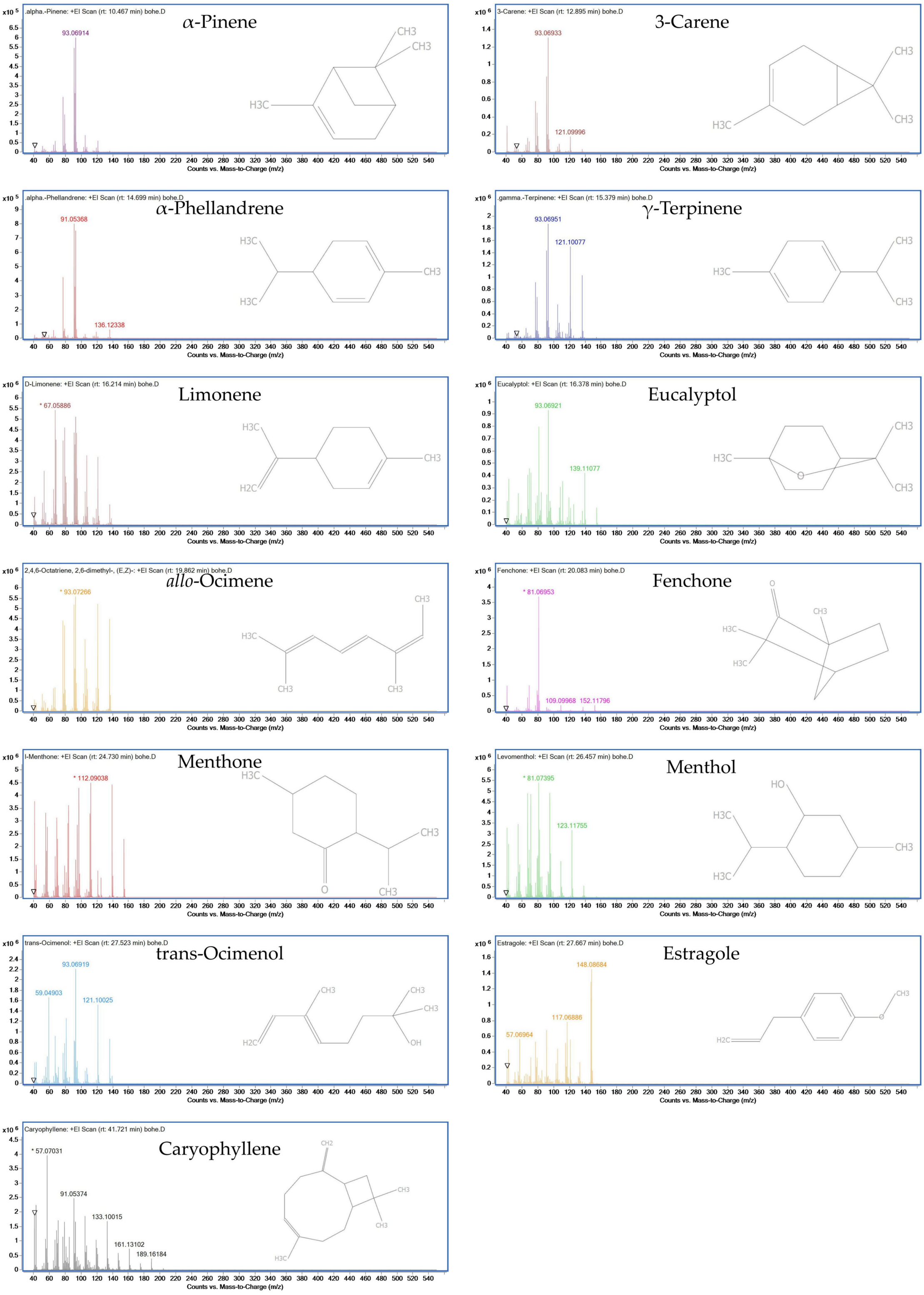

Figure S2. Mass spectra and chemical structures of the 13 VOCs identified through mass spectral library matching and retention index comparison.

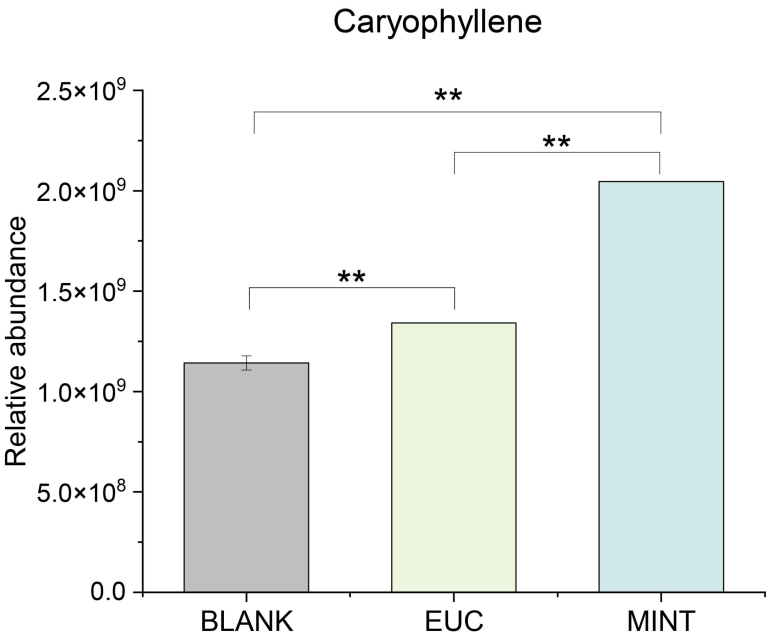

Figure S3. Comparison of relative abundance of caryophyllene among BLANK, EUC, and MINT Samples. ** indicates $p < 0.01$ (Student's t-test)
